# Role of nutraceutical against exposure to pesticide residues: power of bioactive compounds

**DOI:** 10.3389/fnut.2024.1342881

**Published:** 2024-04-17

**Authors:** Mabil Sajad, Shabnam Shabir, Sandeep Kumar Singh, Rima Bhardwaj, Walaa F. Alsanie, Abdulhakeem S. Alamri, Majid Alhomrani, Abdulaziz Alsharif, Emanuel Vamanu, Mahendra P. Singh

**Affiliations:** ^1^School of Bioengineering and Biosciences, Lovely Professional University, Phagwara, India; ^2^Indian Scientific Education and Technology Foundation, Lucknow, India; ^3^Department of Chemistry, Poona College, Savitribai Phule Pune University, Pune, India; ^4^Department of Clinical Laboratory Sciences, The Faculty of Applied Medical Sciences, Taif University, Taif, Saudi Arabia; ^5^Research Center for Health Sciences, Deanship of Graduate Studies and Scientific Research, Taif University, Taif, Saudi Arabia; ^6^Faculty of Biotechnology, University of Agricultural Sciences and Veterinary Medicine, Bucharest, Romania; ^7^Department of Zoology, Deen Dayal Upadhyay Gorakhpur University, Gorakhpur, India; ^8^Centre of Genomics and Bioinformatics, Deen Dayal Upadhyay Gorakhpur University, Gorakhpur, India

**Keywords:** pesticides, nutraceuticals, reactive oxygen species, apoptosis, cytotoxicity

## Abstract

Pesticides play a crucial role in modern agriculture, aiding in the protection of crops from pests and diseases. However, their indiscriminate use has raised concerns about their potential adverse effects on human health and the environment. Pesticide residues in food and water supplies are a serious health hazards to the general public since long-term exposure can cause cancer, endocrine disruption, and neurotoxicity, among other health problems. In response to these concerns, researchers and health professionals have been exploring alternative approaches to mitigate the toxic effects of pesticide residues. Bioactive substances called nutraceuticals that come from whole foods including fruits, vegetables, herbs, and spices have drawn interest because of their ability to mitigate the negative effects of pesticide residues. These substances, which include minerals, vitamins, antioxidants, and polyphenols, have a variety of biological actions that may assist in the body’s detoxification and healing of harm from pesticide exposure. In this context, this review aims to explore the potential of nutraceutical interventions as a promising strategy to mitigate the toxic effects of pesticide residues.

## 1 Introduction

Pesticides have become indispensable tools in modern agriculture, ensuring the protection of crops from pests and diseases, thus securing food production and global food security (1). Nevertheless, there are significant concerns about the unintended effects of pesticides on the environment and human health due to their widespread usage. Among these concerns, the presence of pesticide residues in food and water sources has emerged as a pressing issue, with potentially detrimental effects on both human and ecological systems (2). On the other hand, nutraceuticals have gained significant attention as functional foods that offer potential health benefits beyond basic nutrition (3). Derived from natural food sources, nutraceuticals contain biologically active compounds, such as vitamins, minerals, antioxidants, and phytochemicals, which can promote health, prevent chronic diseases, and enhance overall wellbeing. The increasing demand for nutraceuticals stems from the desire to seek alternative approaches to traditional medicine for maintaining and improving health (4). However, the use of pesticides in conventional agricultural practices poses a significant challenge to the concept of food-based nutraceuticals. Pesticides, despite their intended purpose of safeguarding crops, can leave residues on fruits, vegetables, and other agricultural products. These residues can contaminate the food supply chain, leading to potential health risks for consumers (5).

The presence of pesticide residues in food has raised concerns about their adverse effects on human health. Some pesticides have been linked to various health problems, including developmental disorders, hormonal disruption, neurotoxicity, and even certain types of cancer. Thus, the substances used to protect and enhance food production can inadvertently become a threat to human health (6). In contrast, food-based nutraceuticals offer a promising solution to counterbalance the potential harm caused by pesticide residues (7). These natural compounds found in fruits, vegetables, whole grains, and other food sources possess significant antioxidant and anti-inflammatory properties (8). They can help combat oxidative stress, neutralize harmful free radicals, support immune function, and contribute to overall health and wellness. The key challenge lies in minimizing pesticide residues in food products to ensure the safety and efficacy of nutraceuticals (9). Adopting sustainable agricultural practices, such as organic farming, integrated pest management (IPM), and precision agriculture, can reduce reliance on synthetic pesticides and promote the production of cleaner, pesticide-free crops (10). The use of pesticides in agricultural practices raises concerns about their potential impact on human health and the environment. While pesticide residues can pose risks, food-based nutraceuticals derived from natural sources offer a potential solution to counteract these risks (11). By adopting sustainable agricultural practices and ensuring strict monitoring, it is possible to reduce pesticide residues and promote the production of safe, nutrient-rich crops. Striking a balance between the benefits of pesticides in crop protection and the potential harm they may cause is crucial for ensuring a healthy and sustainable food system (12).

The global pesticide consumption in 2019 was approximately 4.19 million metric tons, where China was by far the largest pesticide-consuming country (1.76 million metric tons), followed by the United States (408 thousand tons), Brazil (377 thousand tons), and Argentina (204 thousand tons) (13). In southeast Asia, the WHO reported an annual increase in pesticide usage, with 20% of developing countries as pesticide consumers, including Cambodia, Laos, and Vietnam. India belongs to one of the major pesticide producing countries in Asia, having 90 thousand tons annual production of organochlorine pesticides including benzene hexachloride (14).

An increase in the usage of pesticides can cause endocrine and neurological disturbances, influencing development, reproduction, and the immune system (15). Pesticides may also influence fundamental regulatory and homeostatic systems, drive reactive oxygen species (ROS)-induced alteration of cellular components, and deplete antioxidant mechanisms, all of which contribute to the occurrence of oxidative stress (16). This review highlights various studies and research that have explored the presence of pesticide residues in food and their potential health implications. It delves into the adverse effects of chronic pesticide exposure, such as neurotoxicity, endocrine disruption, and carcinogenicity, among others. Additionally, it discusses the mechanisms by which pesticide residues can accumulate in the human body and the challenges associated with regulating their usage and monitoring their presence in food.

## 2 Classification of pesticides

Pesticides are categorized according to their toxicological attributes (17). The World Health Organization (WHO) has created a generally accepted pesticide classification system based on acute toxicity values. This method divides pesticides into four categories:

Class I: highly hazardous pesticides. They have high acute toxicity and constitute a serious risk to human health. Organophosphates and carbamates are examples (18).
Class II: moderately hazardous pesticides. They have moderate acute toxicity and constitute a substantial risk to human health. Some pyrethroids and herbicides are examples (19).
Class III: slightly hazardous pesticides. They have modest acute toxicity but can still be dangerous. Fungicides and insecticides are two examples (20).
Class IV: unlikely to present an acute hazard. Pesticides in this class have low toxicity. Some herbicides and insecticides are examples (21).

Here, we discuss that the classification of pesticides as highly hazardous is often based on their acute toxicity, which refers to the immediate detrimental effects that might emerge following short-term exposure. Pesticides designated very toxic can cause serious health problems even at modest levels of exposure (22).

### 2.1 Organochlorine

Organochlorine insecticides are organic compounds that are linked to five or even more chlorine atoms (also known as chlorinated hydrocarbons). They are used in agriculture and were among the first pesticides developed (23). There are two types of organochlorides: DDT-type substances and chlorinated alicyclic substances, comprising bornanes, cyclohexanes, and cyclodienes. Organochlorine is also a broad category of chlorinated hydrocarbons, which also includes chlorinated compounds of hexachlorobenzene (HCB), diphenyl ethane, cyclodiene (aldrin, endrin, and dieldrin), hexachlorocyclohexane (or lindane), nonachloride, chlordane, heptachlor, and heptachlor epoxide (24). Such chemicals have bioaccumulated in nature as a result of their extensive use, lipophilic nature, persistence, and narrow-spectrum action (25). The primary site of action for these substances or their metabolites in humans is the central nervous system, where they alter the electrophysiological characteristics and enzymatic neuronal membranes. These changes in the dynamics of Na^+^ and K^+^ flow through the cell membranes of neurons result in the propagation of multiple nerve impulses for each external stimulus, which can cause symptoms such as seizures and acute respiratory poisoning or even death from respiratory failure (26).

### 2.2 Organophosphates

They are phosphoric acid esters, which are classified as wide-spectrum insecticides because they contain a diverse range of compounds (27). This class of compounds is differentiated by the covalent substitution of one of the four carbon-oxygen-phosphorus links of phosphate ester with the carbon-phosphate bond (28). Organophosphorus insecticides fall under a variety of other chemical categories, such as organothiophosphate, aliphatic amide, aliphatic organothiophosphate, oxime organothiophosphate, benzotriazine organothiophosphate, heterocyclic organiorthophosphate, benzothiopyran organothiophosphate, isoindole organothiophosphate, benzotriazine organothiophosphate, isoxazole organothiophosphate, pyrazolopyrimidine organothiophosphate, pyrimidine organothiophosphate, pyridine organothiophosphate, thiadiazole organothiophosphate, quinoxaline organothiophosphate, triazole organothiophosphate, phosphonate phosphonothioate, phenyl organothiophosphate, phenyl ethylphosphonothioate, phosphoramidate, phenylphosphonothioate, phosphorodiamide, and phosphate oramidothioate (19). Organophosphorus pesticides include mipafox, diazinon, chlorpyrifos, ethion, dichlorvos, malathionparathion, and fenthion. The cholinesterase-inhibiting effects of organophosphorus pesticides on invertebrates and vertebrates result in acetylcholine neurotransmitter superimposition across synapses. Because nerve impulses are unable to cross the synapse, voluntary muscles contract quickly, resulting in paralysis and death (18).

### 2.3 Carbamates

Pesticides made from organic carbamic acid are known as carbamates. These compounds comprise aminocarb, carbaryl, and carbofuran (29). Similar to carbamates, organophosphate pesticides also work by interfering with nerve signal transmission and destroying pests by poisoning them (30). Aldicarb, trimethacarb, carbofuran, carbaryl, propoxur, ethinenocarb, methomyl, oxamyl, pirimicarb, and fenobucarb are common substances that cause hazardous exposure (31).

### 2.4 Pyrethroid

They are synthesized derivatives of natural pyrethrin. Compared to natural pyrethrins, they are more reliable and have prolonged residual effects (32). Although they are moderately poisonous to mammals, they are extremely detrimental to insects. For instance, cyclomethrin and decamethrin come under the pyrethroid category (33).

Overall, the classification of pesticides as highly hazardous serves as a crucial tool for identifying and managing the risks associated with their use. It emphasizes the significance of taking preventative precautions, following correct handling rules, and moving to safer and more sustainable pest management practices (34).

### 2.5 Pesticide exposure

Previous report demonstrated that pesticide exposure contributes to over 300,000 deaths worldwide annually (35). It is characterized by a state in which an individual ingests or inhales any pesticide beyond its threshold levels, which causes adverse effects. Pesticides can penetrate the organism by inhalation, contact with skin, and ingestion. Pesticide exposure can be occupational, as people who work in forestry, agriculture, the pesticide industry, and pesticide application are exposed to pesticides on the job (36). Domestic use (clothing) and dietary intake of tainted food and drink are the sources of nonoccupational exposure, which can lead to significant complications as they are absorbed by the environment and food chain. Although they can be eliminated through the skin, exhaled, and urine, pesticides are transported throughout the body by the bloodstream (37). Pesticides can penetrate the body through the mouth, skin, eye, and respiratory routes, which are the four most prevalent entry points. Pesticides are an essential component of fundamental agricultural processes all over the world, and farmer exposure to them is an inevitable occupational hazard (38). Farmers, according to various studies, are more prone to neurological, digestive, retinal, respiratory, and reproductive disorders than the general population and it is suspected to be influenced by the regular exposure to harmful chemicals but not only, as other factors related to way of life and hard work are also affecting their health (34). Pesticide exposure can cause immediate health problems such as acute poisoning, nausea, eye irritation, pain, and headaches. Diabetes, cancer, asthma, and other medical disorders might develop as a result of prolonged exposure. Pesticide exposure has a negative impact based on the quantity of pesticide used and the period of its retention, i.e., how prolonged it stays there. We are repeatedly exposed to pesticides in our regular activities, either directly or indirectly (39).

## 3 Mechanism of oxidative stress caused by some pesticides

Exposure to pesticides and many chemicals can lead to oxidative stress in living organisms. Oxidative stress occurs when there is an imbalance between the production of ROS and the body’s antioxidant defense mechanisms. Pesticides can induce oxidative stress through various mechanisms (35). Some pesticides (such as rotenone) can directly generate ROS, such as superoxide anion (O_2_^⋅⁣–^), hydrogen peroxide (H_2_O_2_), and hydroxyl radicals (⋅OH). These ROS can damage cellular components, including lipids, proteins, and DNA, leading to oxidative stress. Pesticides can deplete the body’s antioxidant defenses, including enzymes such as superoxide dismutase (SOD), catalase (CAT), and glutathione peroxidase (GPx) (40). These enzymes play a crucial role in neutralizing ROS. Pesticides can inhibit or reduce the activity of these enzymes, impairing the antioxidant defense system. Some pesticide exposure can lead to lipid peroxidation; a process in which ROS attack and damage lipid molecules in cell membranes. This process generates lipid peroxides, which further exacerbate oxidative stress and cause membrane dysfunction. Some pesticides can directly interact with DNA, causing oxidative damage to DNA molecules (41). This can lead to mutations, DNA strand breaks, and impaired DNA repair mechanisms, which contribute to cellular dysfunction and oxidative stress. Some Pesticides can disrupt mitochondrial function, leading to increased ROS production. Mitochondria are a major source of ROS generation in cells. Pesticide-induced mitochondrial dysfunction can impair the electron transport chain, leading to increased electron leakage and ROS production. Pesticide exposure can trigger inflammation in tissues and organs. Inflammatory cells produce ROS as part of the immune response, contributing to oxidative stress. Pesticide-induced inflammation can further promote oxidative damage and disrupt cellular homeostasis (42).

**FIGURE 1:**
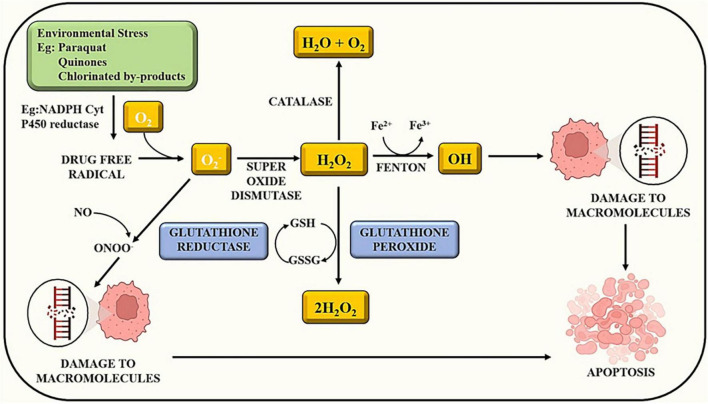
Chemical exposure induces reactive oxygen species and alters other signaling cascades, which results in cell apoptosis.

Exposure to certain chemicals or toxins can initiate a cascade of events within cells. Some compounds have the potential to produce reactive oxygen species. Oxidative stress develops when there is an imbalance between the creation of ROS and the ability of cells to detoxify them. ROS can cause damage to biological structures such as lipids, proteins, and DNA. ROS can disrupt several signaling pathways within the cell. ROS, for example, can trigger signaling cascades involving transcription factors such as NF-B and AP-1, which influence gene expression in inflammation and cell survival (43). Apoptosis, also known as programmed cell death, is a natural mechanism that aids in the elimination of damaged or undesirable cells. Apoptosis can be triggered by excessive oxidative stress and disrupted signaling cascades. This is a defense mechanism designed to keep injured cells from causing more harm as illustrated in Figure 1 (25). Overall, the sequence of events demonstrates the complex link between chemical exposure, oxidative stress, altered signaling, and cellular responses (19). Understanding how pesticides cause oxidative stress can aid in the development of ways to minimize their adverse effects and encourage safer pesticide use (Table 1).

**TABLE 1:** Some examples of well-known pesticides and their oxidative stress response and related toxicity.

S No.	Name of pesticides	Chemical structure	Used for crop	Cellular responses	References
1	Triflorin	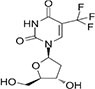	Glycine max, citrus, and *Coffea arabica*	It can induce microtubules in *Oreochromis niloticus* and is genotoxic to mice and *Drosophila melanogaster*. In humans it is fighting leishmaniasis.	(44)
2	Parathion	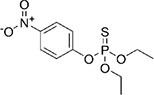	Barley, canola, corn, cotton, sorghum, sunflowers, and wheat	It causes immunotoxic reactions such as enhanced *in vitro* lymphocyte apoptosis, lowers IgM and germ cells center-positive B-lymphocyte numbers as well as antigen-specific immunotoxicology IgM responses.	(45)
3	DDT	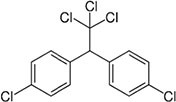	Beans, sweet, potatoes, peanuts, cabbage, tomatoes, and cauliflower	In both humans and laboratory animals, dichlorodiphenyltrichloroethane (DDT) and its metabolite dichlorodiphenyldichloroe-thylene (DDE) have been linked to an increased risk of insulin resistance and obesity.	(46)
4	Dieldrin	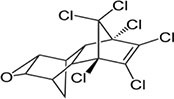	Corn, cotton, and citrus fruit	Through a nuclear receptor CAR-mediated method of action, it causes liver cancers in mice.	(47)
5	Mancozeb	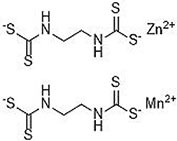	Tree fruits, vegetables crops, field crops, nuts, and ornamentals	In mice exposed to (mancozeb) MCZ, the ovarian structure was harmed, and apoptosis was elevated. MCZ can affect the abnormal function of mitochondrial respiratory chain, lead to oxidative phosphorylation decoupling, produce oxidative stress, and finally cause ovarian injury and apoptosis in mice.	(48)
6	Endosulfan	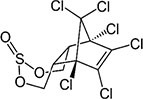	Soybean	Endosulfan was found to cause DNA damage in grass carp (*Ctenopharyngodon idella*).	(49)
7	Monocrotophos	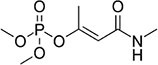	Maize, rice, sugarcane, cotton, groundnut, soybeans, and vegetables	In nematodes (*Caenorhabditis elegans*) and arthropods (*Culex quinquefasciatus*), causes paralysis, acetylcholine inhibition, increased micronuclei, and a decrease in the DNA/RNA ratio.	(50)
8	Carbofuran	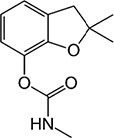	Potatoes, corn, soybeans, strawberries, grapes, wheat, and alfa-alfa	Because to its anticholinesterase function, which blocks the action of acetyl-cholinesterase and butyrylcholines, it is exceedingly deadly to animals, birds, fishes, and other species. Carbofuran is linked to endothelial dysfunction, anomalies in the reproductive cycle, and cytotoxicity and mutagenicity effects in humans.	(51)
9	Pendimethalin	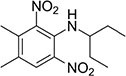	Cereals, legumes, and vegetable crops	ROS production, apoptosis induction, and DNA damage were all detected in rat bone marrow and human lymphocytes.	(52)
10	Cyhalothrin	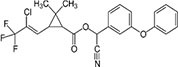	Cotton, cereals, hops, ornamentals, potatoes, and vegetables	It binds to the voltage-gated sodium channel in nerve and tremors in poisoned insects. Due to this insect lose control on nervous system.	(53)
11	Phorate	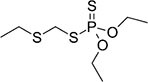	Corn, sugar beets, cotton, coffee, potatoes, beans, wheat, some ornamental peanuts, herbaceous plants, and bulb	It is observed in comet assay that the exposure of phorate to human lymphocytes results in DNA damage.	(54)
12	Captan	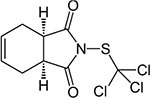	Apples, peaches, strawberries, almonds. Ornamental plants, turf, and seeds	Apoptotic and necrotic cell death caused by captan, it increases the intrinsic Ca^2+^ and Zn^2+^, also increases hydrogen peroxide cytotoxicity and reduce the levels of cellular thiol compounds prior to cell death.	(55)
13	Isoproturon	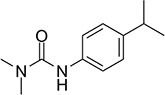	Wheat, cereal, sugarcane, citrus, cotton, and asparagus	Isoproturon boosted kidney and heart weight while decreasing epididymis weight. Organ histopathological changes were dose dependent.	(56)
14	Dichlorvos	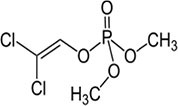	Cotton, coffee, tea, and cocoa	Elderly male Swiss mice exposed to dichlorvos experience reactive oxygen species generation, altered gene expression for acetylcholinesterase (AChE), and significantly higher levels of protein carbonyl content (PCC) and thiobarbituric acid reactive substances.	(57)
15	Quinalphos	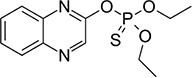	Wheat, rice, coffee, sugarcane, and cotton	Low expression of antioxidant biomarkers in the kidney tissue of wistar rats, which include total thiol groups, glutathione s-transferase, superoxide dismutase, glutathione peroxidase, catalase, and glutathione reductase, as well as enhanced thiobarbituric acid reacting substance levels, suggested that considerable oxidative damage to the renal tissue happened after multiple doses of quinalphos.	(58)
16	Cypermethrin	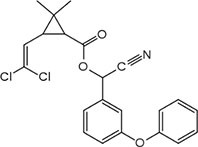	Cotton and fresh vegetables	In zebra fish, it has been found that there is a decrease in egg production, a delay in the growth of gonadotropins, significant changes in the levels of plasma 17-estradiol (E2) and testosterone, disruption of the release of sex hormones, and anomalies in the gene expression of the thalamic axis.	(59)
17	Spinosad	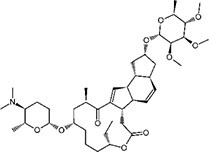	Cotton, corn, soybeans, and tomato	Due to Spinosad injection inhibition of acetylcholinesterase in the liver and brain of *Oreochromis niloticus*. Spinosad induces fast stimulation of the organism’s nervous system by stimulating ɣ-aminobutyric acid (GABA) and nAChR receptors, resulting in paralysis and death.	(60)
18	Azadirachtin	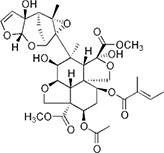	Tomatoes, cabbage, potatoes, cotton, tea, tobacco, coffee, and protected crops	In silkworm, the Sf9 cell line’s Ca^2+^ Mg^2+^ATPase activity was significantly increased by azadirachtin, indicating that the calcium signaling pathway may be involved in growth control and the process of death in the prothoracic gland.	(61)
19	Pyrethroid	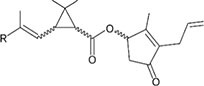	Cotton, tea, and vegetables	Pyrethroid produce oxidative damage in fish species such as tilapia, carp, and trout through ROS-mediated mechanisms.	(62)
20	Bifenthrin	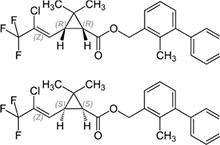	Bananas, apples, pears, and ornamentals	Bifenthrin has the capacity to cause severe oxidative stress (lipid peroxidation) in the kidney, lung, and liver of rats and in insect *Spodoptera frugiperda*, it causes DNA damage and autophagy, and reduces viability of Sf9 cells.	(63, 64)

In contrast, high ROS concentrations cause cellular biomolecule damage, which results in the emergence of numerous illnesses (65). The human body contains a number of defense systems to mitigate the harm caused by oxidative stress. Antioxidative agents’ action is the primary mechanism (66). Since they are stable enough to eliminate free radicals by donating electrons, antioxidants are molecules that prevent or limit oxidative injury (67). Many substances have recently been discovered to have antioxidative properties, but the human body has two types of antioxidant defense systems: enzymatic and nonenzymatic. By preserving a physiologic amount of ROS, they defend both cellular structural integrity and performance from ROS harm (68). The enzymatic antioxidants include catalase (present in peroxisomes), glutathione peroxidase, which contains selenium (Se-GPX) and resides in the matrix of mitochondria and cytosol, and glutathione reductase (GSH-R), which is considered a key enzyme for the glutathione (GSH) redistribution pathway. Copper and zinc-containing SOD (Cu, Zn-SOD) is present in peroxisomes and CAT. These factors maintain the cellular homeostasis of the human body (54).

Elevated levels of oxidative stress and endocrine disruption have been linked to pesticide actions in the human body. Exposure to pesticides is a major factor in the rise in oxidative stress, which might change a person’s susceptibility to diseases. They have been discovered in the human placenta and have been linked to increased oxidative stress, intrauterine limitation, and decreased birth weight. In particular, lipid peroxidation (one of the key oxidative stress markers) and cytotoxicity have been observed in humans, primarily in pregnant women and agricultural producers (69). However, studies utilizing pesticides on mammals that directly connect the lethality of the contaminants and oxidative stress are exceedingly rare. As a result, research employing human cell lines started, and it is now being done more often. The effects of pesticides on the extent of oxidative stress in human tissues and changes in the endocrine system were demonstrated using human cell cultures. The precise action mechanisms of phenoxy analogs, triazolo pyrimidines, triazines, dinitroanilines, imidazolines, and other types of fungicides and insecticides are typically difficult to pinpoint. Particularly in cases where the effect is brought on by a dose of environmental exposure, these pathways in humans are not well understood (70).

As previously noted, in human cell lines, the oxidative stress variables under the action of pesticides were studied. It was discovered that the herbicide MCPA (4-chloro-2-methylphenoxyacetic acid) is carried across the membranes of Caco-2 cells, which is a useful study model of the human digestive tract. Caco-2 cells cultivated on semipermeable membranes were utilized to examine and characterize the mechanisms of transcellular transport of MCPA across the small intestine, where pesticides are absorbed (71). Another study looked at the impact of the sodium salts of 2,4-D-Na and 4-chloro-2-methylphenoxyacetic acid (MCPA-Na), two extensively used phenoxy herbicides, on the amount of carbonyl groups in protein substrates in erythrocytes. Because of their biological and structural simplicity, making them particularly suited for research on specific xenobiotic toxicity, human erythrocytes were chosen as such an experimental animal model (68). The results of the experiments showed that the location of the hydroxyl in the phenol ring has a significant impact on the pro-oxidative activity of phenoxy herbicides. According to previous literature, paraquat (1,1-dimethyl-4,40-bipyridinium chloride), one of the most commonly used herbicides, causes mitochondrial dysfunction in human bronchial BEAS-2B cells. Because these cells were developed from normal healthy bronchi, they are a suitable model system to investigate the pathogenic mechanisms of oxidative stress-induced respiratory ailments caused by pesticides. Furthermore, the mechanisms behind oxidative stress are frequently studied using this cell line (72).

## 4 Signaling mechanism related with oxidative stress in some pesticides

Although some pesticide exposure is acute or chronic, a number of events comprising various cellular pathways, including alterations in gene activation, expression, or suppression, take place (73). The exact molecular mechanism by which this happens is still unknown. Understanding the molecular and cellular level alterations is essential for determining the primary pathways associated with the induction of oxidative stress due to some pesticides and evaluating potential defensive medications or therapies (74).

### 4.1 TNF-α pathway

According to several investigations, one of the potential mechanisms responsible for oxidative stress is the death receptor pathway (75). The signaling of caspase-8, which catalyzes the breakdown of the caspase-3 effector either indirectly or directly through the mitochondrial pathway, is brought on by the conjugation of cell surface death receptors, including the tumor necrosis factor receptor (TNFR), which facilitates the transmitted signal of tumor necrosis factor-alpha (TNF-α). TNF- is a potent pro-inflammatory cytokine that is produced during inflammation by macrophages and monocytes and is in control of many cell signaling pathways that trigger necrosis or apoptosis (76). The association between TNF-α and the two cell surface receptors TNF-R1 and TNF-R is what causes the inflammatory responses that it triggers. TNF-α is also responsible for the activation and transcription of adhesion molecules, the stimulation of inflammatory cytokines, and the promotion of growth. The mouse fibrosarcoma cell line (L929) could experience mitochondrial dysfunction and ROS production as a result of increased TNF-α levels alone (77). Based on an experiment on mice exposed to permethrin, a rise in TNF- levels induces ROS generation and reduces the redox equilibrium, which causes oxidative stress (78). Permethrin also enhanced TNF-mRNA expression in zebrafish exposed for a period of 72 h after fertilization in a concentration-dependent manner (65).

### 4.2 NF-κB and Nurr1 pathway

The transcription factor orphan nuclear receptor-related 1 (Nurr1), a constituent of the nuclear receptor subclass 4 group A member 2 (NR4A2) family of proteins, is crucial for dopaminergic neuron metabolism. New research suggests that decreased Nurr1 activity may play a role in Parkinson’s disease etiology. In brain cells, Nurr1 prevents NF-B (transcription factor NF-B), which has anti-inflammatory properties (79). The pro-inflammatory nuclear factor-B, i.e., transcription factor, is expressed more, while the Nurr1 gene expression is lowered by permethrin (80). Permethrin (insecticide) enhanced the production of the pro-inflammatory cytokine TNF- and inhibited the expression of IL-13, IL-2, and IL-1 in an additional investigation in older treated rats. These findings suggest that the Nurr1, NF-κB, and TNF-α pathways may contribute to understanding some pathways associated with oxidative stress caused by pesticides (81).

### 4.3 STAT

**FIGURE 2:**
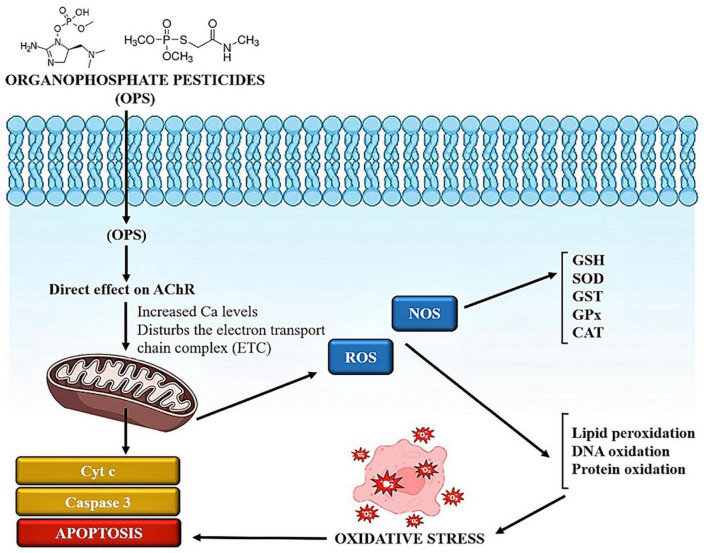
The role of oxidative stress in pesticide-induced toxicities.

According to a plethora of studies, chlorpyrifos (CPF) weakens antioxidant defenses by interfering with the electron transport chain in mitochondria (ETC) complex 1 activity, which increases the formation of free radicals and superoxide. According to an earlier investigation, CPF increased cell mortality in a dose-dependent manner and caused oxidative stress by increasing ROS production and lowering GSH levels in dopaminergic neurons and human mesencephalic cells (82). The team hypothesized that signaling transducers and activators of transcription 1 (STAT-1) regulate CPF-induced ROS production and that higher ROS ultimately result in cell apoptosis based on the findings of CPF-induced dopaminergic cell damage (Figure 2). Apoptotic cell mortality subsequently results from increased reactive oxygen species. STAT proteins are members of a group of latent cytoplasmic transcriptional regulators that are crucial for cell type-specific differentiation, growth, apoptosis, and multiplication. Both neuronal viability and cell death depend on Janus kinase (JAK)-STAT signaling (83). The activation of JAK that results from the binding of mediators to their receptors phosphorylates STAT-1 on serine 727 and tyrosine 701 residues. Phospho STAT-1 dimerizes and moves into the nucleus, where it attaches to the GAS/ISRE found on the coding region of particular target genes that control NADPH oxidase (NOX), pro-inflammatory cytokines, apoptosis, and regulators of cell cycle arrest such fas, caspases, and bax. Proapoptotic gene transcription-dependent expression as well as non-transcriptional signal transduction pathways are regulated by STAT-1 to control apoptosis. In yet another study, when organophosphate pesticides (OP) pesticides were used on STAT-1 suppression (KD) dopaminergic cells, they caused a 66% reduction in mitochondrial ROS levels compared to scrambled tiny interfering RNA (siRNA)-transfected cells subjected to identical pesticides (84). NOX-1, an NADPH oxidase isoform that produces superoxide, controls the production of ROS in a variety of cell types, including but not limited to monocytes, macrophages, vascular endothelial cells, and smooth muscle cells. The primary ROS-producing enzyme throughout inflammation is NOX1. The fact that OP pesticides boosted the recruitment of STAT-1 to the constitutive NOX1 promoter suggests that STAT-1 is responsible for controlling the transcription of NOX1. In neural cells exposed to the OP pesticide CPF, STAT-1 is critical in controlling ROS production as well as GSH levels in a NOX1-dependent manner. In human monocytes, organochlorine pesticides increased NOX-dependent ROS production. These findings collectively imply that NOX-STAT-1 activation is critical for the production of ROS during OP pesticide-induced oxidative damage (85).

### 4.4 Endoplasmic reticulum stress

Assembly, folding, posttranslational alteration, and protein transport are only a few of the many tasks performed by the endoplasmic reticulum (ER). Additionally, calcium, which is necessary for muscular contractions, is stored in the ER. When the ER’s (86) aptitude for folding peptides is exceeded, ER stress results, and cells with ER stress are characterized by a build-up of abnormal proteins within the ER lumen. Numerous factors, such as hypoxia, nutritional scarcity, and pesticides, can cause ER stress. Cellular senescence might be produced in the event that ER stress is substantial or prolonged. It has been demonstrated that a number of pesticides, including 2,4-dichlorophenol, paraquat, chlorpyrifos, and deltamethrin, cause ER stress. Numerous of these pesticides also cause apoptosis, although research indicates that ER stress and apoptotic cell death are caused by separate chemical mechanisms (78).

### 4.5 Mitochondrial dysfunction pathway (mitochondrial apoptosis)

Mitochondrial dysfunction is a typical oxidative stress-related malfunction. ROS can induce mitochondrial damage in some circumstances, but they can also cause mitochondrial damage in other circumstances. Numerous pesticides have been found to block mitochondrial ensembles, which are the primary site for ROS generation (87). As a result, it is believed that the ROS generated by damaged mitochondria play a significant role in oxidative stress. In both mammals and fish, ER stress and apoptosis are frequently linked to mitochondrial malfunction (65). Pentachlorophenol (PCP) and its metabolite tetrachlorohydroquinone (TCHQ) massively increased lipid oxidation in the rat liver and lowered the antioxidant GSH level by producing large amounts of urine 8-iso-prostaglandin-F2 (8-iso-PG-F2). The mitochondrial apoptotic pathway is another possible mechanism that may be implicated in pesticide such as atrazine (ATR), organophosphorus (OP) compounds, carbamates, and pyrethroids induced mitochondrial dysfunction, according to the available and developing evidence (88). The primary regulators of mitochondrial coherence in this system are B-cell lymphoma 2 (BCL2) and BCL2-associated X (BAX). They also affect the release of cytochrome c and the activation of caspase. Several well-known proteins linked to cell damage, BAX and BCL2, have opposing roles. In contrast to the BAX peptide, which acts as an apoptosis activator, the BCL2 protein suppresses apoptosis by its own antioxidative action (89). Following mitochondrial injury, Bax is translocated from the cytoplasm to the mitochondria, and Bcl-2 expression is significantly reduced. Mitochondrial cytochrome c is released into the cytoplasm, which is a crucial terminal event, as a result of high amounts of ROS from exposure to pesticides. TCHQ increased the transcription of Hsp70 in hepatocytes while decreasing the expression of the BCL2/BAX ratio and cellular apoptotic sensitivity (CAS), two genes involved in apoptotic and necrotic processes (90).

## 5 Relationship of the toxicity of some pesticides with alteration of oxidative balance

### 5.1 Neurotoxicity

Neurotoxins are any compounds that can damage the central nervous system, along with the brain (80). The brain is particularly prone to OP toxicity, which disrupts the balance of oxidant and antioxidant components in neuronal cells (91). OPs have a strong pro-oxidant action that disrupts neuronal mitochondrial function, resulting in a variety of neurological disorders. Higher level exposures from occupational exposures and closeness to agricultural spraying are linked to a variety of neurotoxicity in people (92).

Human neurodegenerative disorders and neurotoxicity have been associated with pyrethroid pesticides, organochlorine insecticides, and organophosphate insecticides. DDT has low toxicity (rat oral LD_50_ = 113 mg/kg) (93). Furthermore, oral administration of 3.5 or 35 mg DDT/day to people for up to 18 months did not result in observable toxicity or neurotoxicity. Only a few human studies have examined DDT’s possible neurotoxicity. DDT was identified more frequently in the brains of Alzheimer’s sufferers. Nonoccupational DDT exposure can potentially result in cognitive impairments (94). Dieldrin caused substantial oxidative stress, mostly owing to mitochondrial malfunction, and was associated with considerable caspase overexpression and activation, resulting in apoptosis (95). Dieldrin-induced dopaminergic neurotoxicity is caused by caspase-3-dependent proteolytic cleavage of protein kinase C (PKCδ) (96). Dieldrin has also been shown to cause epigenetic dysregulation in cell and animal models of dieldrin neurotoxicity by hyperacetylation of core histones. Endosulfan is very neurotoxic in rats, inducing both chronic neurodegeneration and developmental neurotoxicity.

Aside from inducing apoptotic cell death through mitochondrial dysfunction. According to research (97), rats gavaged with 2 mg/kg endosulfan for 6 days had damaged brain mitochondria, as evidenced by significantly lower levels of SOD, GSH, and CAT, as well as enhanced lipid peroxidation.

### 5.2 Disruption of the endocrine system

Every hormone has a different molecular structure and a unique production process with numerous phases. The production of the hormone may be impaired or the characteristics of the hormones may change if one component or link in the chain of hormone synthesis is disrupted. Some pesticides, including prochloraz, fenarimol, and other fungicides (imidazole), have the capacity to block testosterone to oestrogen conversion by inhibiting cytochrome 19 aromatase *in vitro* (98). It was predicted that substances that may decrease the activity of aromatase *in vitro* might also be able to alter local oestrogen and androgen levels *in vivo*. Aromatase induction is a biological method of xenobiotic deactivation that does not typically result in toxicity. *In vitro* aromatase function is induced by the pesticides simazine, atrazine, and propazine. It has been proven that p,p-DDE induces aromatase both *in vivo* and *in vitro*. Additionally, the pesticide heptachlor may operate as an inducer of testosterone 16-alpha and 16-beta hydroxylases, although pirimicarb, methomyl, iprodione, and propamocarb can only slightly stimulate aromatase activity (99). Dopamine-beta-hydroxylase activity has been demonstrated to be suppressed by thiram, sodium N-methyl-di-thio-carbamate (SMD), and other di-thio-carbamates, which results in a reduced conversion of dopamine to norepinephrine. This might affect the hypothalamic catecholamines that produce the proestrus surge in luteinizing hormone, which triggers the last stages of ovulation. It was determined that SMD has the ability to prevent rat ovulation and the LH surge. Ketoconazole blocks the synthesis of progesterone as well as a number of cytochrome 450-dependent monooxygenase enzymes (100).

### 5.3 Carcinogenicity

The large percentage of pesticides poses a risk to nontarget organisms, including humans. In laboratory animals, 56 pesticides have been identified as cancer-causing. In clinical studies, chemicals such as lindane, phenoxy acid herbicides, toxaphene, and several organophosphates have been linked to cancer (101). Due to the destructive effects of genotoxic substances, cellular genetic components (DNA and RNA) lose their genotoxic characteristics.

It has been studied *in vivo*, *in vitro*, and epidemiologically that certain pesticides can cause genomic toxicity (102). This genotoxicity is viewed as a fundamental health concern that, with repeated exposures, will have long-term consequences on reproductive, neurological, and cancerous systems. Pesticide use might cause mutagenic and non-mutagenic processes that cause genetic changes. Several studies have revealed a strong link between chronic pesticide exposure and a few proto-oncogenes in populations exposed owing to pesticide cytogenetic effects (103). It is linked to an increased risk of leukemia and non-lymphoma, Hodgkin’s as well as cancers of the brain, skin, thyroid, esophagus, kidney, lung, prostate, testicles, cervix, bladder, and rectum (104). These may cause oxidative stress, which results in the production of free radicals and changes in enzymatic antioxidants such as catalase, glutathione transferase and SOD (105).

## 6 Nutraceuticals and bioactive compounds from medicinal plants

Medicinal plants have traditionally been utilized by humans as a traditional way of treating a variety of diseases. Despite significant improvements in therapeutic development, there is still a need for effective and potent analgesic drugs (106). In this context, it has been widely described that numerous plant-derived compounds serve an essential part in the process of developing novel techniques to treat various diseases. Recent research on herbal plants or medicine has resulted in significant advances in the pharmacological assessment of diverse plants utilized in traditional medical systems (107). Secondary metabolites or substances found in medicinal plants include tannins, terpenoids, alkaloids, and flavonoids, which determine the therapeutic effectiveness of the plant, particularly its antioxidant activity. Secondary metabolites are very diverse low molecular weight substances with a wide range of biological characteristics that interact with proteins, nucleic acids, and other bio membranes and are active and volatile targets of cells (108).

Herbal remedies play a crucial part in healthcare and have various therapeutic applications. Here are some key reasons highlighting the importance of medicinal plants and their therapeutic applications:

### 6.1 Traditional medicine

For ages, medicinal plants have been employed in traditional medical systems including Ayurveda, Traditional Chinese Medicine, and Indigenous healing practices. They form the foundation of these systems and have been trusted for their healing properties by communities worldwide (109).

### 6.2 Rich source of bioactive compounds

Medicinal plants contain numerous bioactive compounds including flavonoids, alkaloids, terpenoids, and tannins, which possess medicinal properties. These compounds can have diverse impacts on the human health, including analgesic, anti-inflammatory, antimicrobial, antioxidant, anticancer, and antidiabetic activities (110).

### 6.3 Drug discovery and development

Many modern pharmaceutical drugs are derived from or inspired by medicinal plants. Natural plant substances serve as the foundation for the creation of novel drugs. Examples include the use of the plant-derived compound paclitaxel for cancer treatment and the development of aspirin from willow bark (111). Medicinal herbs are often the primary source of healthcare in many developing countries, where access to conventional medicine may be limited. Local communities rely on traditional herbal remedies derived from medicinal plants for treating common ailments and maintaining their wellbeing (112). Medicinal plants are an integral part of complementary and alternative medicine practices. Many people seek natural and plant-based treatments as alternatives or supplements to conventional medicine. Herbal remedies and supplements made from medicinal plants are used for various purposes, including immune support, stress reduction, and overall wellness (113, 114).

### 6.4 Nutraceuticals and functional foods

Medicinal herbs are also used to create functional meals and nutraceuticals. These products go beyond basic nourishment to give additional health advantages. They are created with specialized plant extracts or bioactive chemicals to promote various areas of health, such as cardiovascular health, cognitive function, and digestive wellness (115, 116).

## 7 Protective effects of various nutraceuticals against some pesticide-induced injuries

**FIGURE 3:**
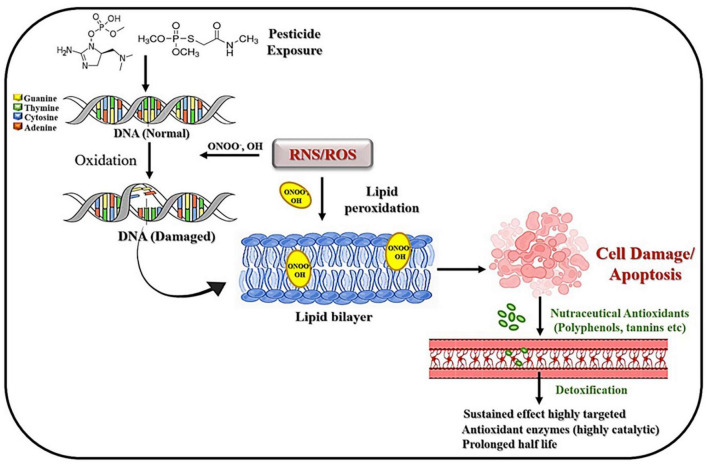
Schematic representation of the interplay between pesticide exposure and cellular responses, including DNA damage, lipid peroxidation, and cell apoptosis, alongside the protective effects of nutraceutical antioxidants through detoxification pathways.

Various nutraceuticals have been studied for their potential protective effects against pesticide-induced toxicities. These natural compounds derived from food sources can possess antioxidant, anti-inflammatory, and detoxifying properties, which may help mitigate the harmful effects of some pesticides (Figure 3) (117).

Nutraceuticals rich in antioxidants, such as vitamins C and E, beta-carotene, and flavonoids, can counteract oxidative stress induced by pesticides. Examples include berries (blueberries and strawberries), citrus fruits, leafy greens (spinach and kale), and cruciferous vegetables (broccoli and cauliflower).

### 7.1 Curcumin

Curcumin, a bioactive component found in turmeric, has been examined for its possible protective mechanisms against pesticide-induced damage (118). While the particular methods may differ based on the pesticide and the target organ or system. Curcumin is a powerful antioxidant that can neutralize the damaging free radicals produced by pesticides (119). This helps to protect cells and tissues from oxidative damage and decreases inflammation (120). Curcumin has the ability to suppress a variety of inflammatory processes in the body. Pesticides can cause inflammation, and curcumin’s anti-inflammatory qualities may assist to mitigate this impact. It may increase the activity of detoxification enzymes in the liver, such as glutathione S-transferases (GSTs), which are important in metabolizing and removing pesticides. Pesticide poisoning frequently targets the liver. Curcumin may help protect the liver from pesticide-induced damage by lowering oxidative stress and inflammation. Curcumin’s anti-apoptotic characteristics may aid in the prevention or reduction of cell death caused by pesticide exposure (121). Some pesticides have been related to an increased cancer risk. By suppressing tumor development and metastasis, curcumin’s anticancer effects may help lessen the carcinogenic risk of some pesticides. Curcumin may boost the generation and activity of natural antioxidant enzymes including SOD and catalase, boosting the body’s ability to counteract pesticide-induced oxidative stress. Curcumin can improve gut health by encouraging the development of good gut bacteria and decreasing inflammation in the gastrointestinal system. This can help reduce some of the negative effects of pesticides on the digestive system (122). A recent study demonstrated that rats treated with mancozeb alone exhibited significant liver damage, characterized by elevated levels of liver enzymes [aspartate transaminase (AST) and alanine transaminase (ALT)] and histopathological alterations indicative of hepatotoxicity. In contrast, rats co-administered with curcumin and mancozeb showed a significant reduction in liver enzyme levels and less severe histopathological changes, indicating the protective effects of curcumin against pesticide-induced hepatotoxicity (88).

### 7.2 Resveratrol

Resveratrol is a polyphenolic chemical found naturally in foods such as red grapes, red wine, and some berries. Its possible preventive mechanisms against pesticide-induced damage have been investigated. Resveratrol is a powerful antioxidant that may neutralize pesticide-generated free radicals. This helps to minimize oxidative stress and tissue damage. Resveratrol has the ability to suppress a variety of inflammatory pathways in the body (123). Pesticides can cause inflammation, which resveratrol’s anti-inflammatory qualities may help to mitigate. Resveratrol may increase the activity of liver detoxification enzymes such as GSTs. These enzymes are essential for pesticide metabolization and elimination. Pesticide poisoning frequently affects the liver. Some pesticides have the ability to cause cell death in diverse tissues. The anti-apoptotic characteristics of resveratrol may help prevent or minimize cell death in response to pesticide exposure (124). Resveratrol has been explored for its neuroprotective properties. It may help protect nerve cells from pesticide damage, potentially lowering the likelihood of neurodegenerative consequences. Some insecticides may be harmful to the cardiovascular system. The cardiovascular advantages of resveratrol, such as its capacity to enhance blood vessel function and lower inflammation, may protect against these consequences. Resveratrol has been linked to anticancer activity. By suppressing tumor development and metastasis, it may help lessen the carcinogenic risk of some pesticides. Resveratrol may promote metabolic health by regulating blood sugar levels and improving lipid profiles. These advantages may assist in mitigating some pesticide-induced metabolic abnormalities. Some pesticides have the potential to damage the endocrine system. The ability of resveratrol to promote hormonal homeostasis may assist to mitigate these abnormalities (94). Kumar et al. (119) provides evidence supporting the potential neuroprotective effects of resveratrol against pesticide-induced neurotoxicity in rats. The findings suggest that resveratrol may offer therapeutic benefits in mitigating the adverse neurological effects associated with pesticide exposure (125).

### 7.3 Flavonoids and flavone glycosides

Flavonoids and flavone glycosides constitute a diverse class of phytochemicals known for their potent antioxidant and anti-inflammatory properties. These compounds have demonstrated significant potential in preventing oxidative damage induced by pesticides. Quercetin is a flavonoid, which is a polyphenolic component present in a variety of fruits, vegetables, and cereals (126, 127). Quercetin is a powerful antioxidant that may neutralize pesticide-generated free radicals. This helps to minimize oxidative stress and tissue damage (128). Histopathological analysis of brain tissues revealed that rats treated with chlorpyrifos alone displayed neuronal damage and gliosis, whereas rats co-administered with quercetin and chlorpyrifos showed preservation of neuronal morphology and reduced gliosis, supporting the neuroprotective effects of quercetin. Rutin is well-known for its antioxidant properties. Pesticides can cause free radicals and oxidative stress in the body, which can lead to cell damage and inflammation (129, 130). Organophosphate insecticides, for example, cause toxicity by blocking cholinesterase enzymes. The ability of rutin to inhibit these enzymes may give protection against pesticide-induced toxicity (131). A recent study measured levels of pro-inflammatory cytokines (such as TNF-α and IL-6) and apoptotic markers (such as caspase-3 activity) in liver and kidney tissues. Rats treated with deltamethrin alone showed increased levels of pro-inflammatory cytokines and caspase-3 activity, indicative of inflammation and apoptosis. However, rats co-administered with rutin and deltamethrin exhibited reduced levels of pro-inflammatory cytokines and caspase-3 activity, suggesting the anti-inflammatory and anti-apoptotic effects of rutin (132). Flavonols like kaempferol may be found in a variety of fruits and vegetables. It is useful in preventing oxidative stress brought on by pesticides because of its potent anti-inflammatory and antioxidant qualities. A prevalent flavone in celery, parsley, and chamomile tea is apigenin. By scavenging free radicals and adjusting the activity of antioxidant enzymes, it has been demonstrated to mitigate oxidative damage caused by pesticides (133).

### 7.4 N-acetylcysteine

N-acetylcysteine (NAC) is an amino acid supplement and pharmaceutical renowned for its mucolytic and antioxidant characteristics. NAC is a precursor of glutathione, one of the most essential antioxidants in the body. NAC can help neutralize damaging free radicals produced by pesticides by raising glutathione levels, lowering oxidative stress and cellular damage (134). NAC helps the body’s detoxification functions, notably in the liver. It can aid in increasing the liver’s ability to metabolize and remove pesticides and other pollutants. NAC is widely used to treat acetaminophen (paracetamol) overdoses. While not specifically related to pesticide toxicity, it demonstrates NAC’s potential to combat toxic chemicals by increasing detoxification mechanisms in the liver. In situations of pesticide inhalation, NAC’s mucolytic characteristics can assist lower the thickness of mucus in the respiratory system, making hazardous chemicals easier to remove from the lungs (135). NAC may have anti-inflammatory characteristics that can aid to reduce the inflammatory response caused by some pesticides. NAC may affect the immune system, perhaps improving the body’s immunological response against pesticide-induced illnesses or inflammatory processes. NAC may protect against the negative effects of pesticides on several organs, including the liver, kidneys, and nervous system, by lowering oxidative stress and oxidative damage in cells and tissues. NAC has been examined for its possible neuroprotective benefits, and it may help protect nerve cells against pesticide damage, lowering the likelihood of neurodegeneration (136). A report assessed biochemical markers of oxidative stress and neuroinflammation in the brain tissues of rats. Rats treated with chlorpyrifos alone showed increased levels of oxidative stress markers (such as malondialdehyde) and pro-inflammatory cytokines (such as TNF-α and IL-1β) compared to control rats. In contrast, rats co-administered with NAC and chlorpyrifos exhibited reduced levels of oxidative stress markers and pro-inflammatory cytokines, indicating the antioxidant and anti-inflammatory properties of NAC (137).

### 7.5 Omega-3 fatty acids

Omega-3 fatty acids, polyunsaturated fats present in foods such as fatty fish, flaxseeds, and walnuts, have been investigated for their possible protective mechanisms against pesticide-induced toxicity. Anti-inflammatory properties of omega-3 fatty acids are well established. Pesticides, notably organophosphates and pyrethroids, have the potential to cause inflammation in the body (138). Omega-3 fatty acids can help decrease inflammation, potentially reducing pesticide harm. Omega-3 fatty acids have antioxidant qualities, which means they can help neutralize the damaging free radicals produced by pesticide exposure. Some research suggests that omega-3 fatty acids can boost the activity of detoxification enzymes in the liver, such as cytochrome P450 enzymes. These enzymes are in charge of breaking down and removing numerous poisons, including pesticides, from the body. Omega-3 fatty acids may alter the immune system’s response to pesticide exposure. They may help the body deal with the harmful effects of pesticides by altering immunological function. Pesticides, particularly some organophosphates, can be neurotoxic (139). Docosahexaenoic acid (DHA), in particular, is critical for brain health and development. They may provide protection against pesticide-induced nervous system harm. Cell membranes rely heavily on omega-3 fatty acids. They can assist preserve the fluidity and integrity of cell membranes, which may protect cells against pesticide-induced disruption. Pro-inflammatory cytokines, which are chemicals involved in the inflammatory response, have been demonstrated to be reduced by omega-3s. This decrease in cytokine production may aid in reducing the inflammatory response caused by certain pesticides (140). The study evaluated the neurobehavioral effects of organophosphate pesticide exposure and the potential protective effects of omega-3 fatty acids using various behavioral tests, including the Y-maze test and the elevated plus maze test. Rats treated with organophosphate pesticide exhibited impaired spatial memory and increased anxiety-like behavior compared to control rats. However, rats co-administered with omega-3 fatty acids and pesticide showed significant improvements in spatial memory and reduced anxiety-like behavior, suggesting the neuroprotective effects of omega-3 fatty acids against pesticide-induced toxicity (141).

### 7.6 Vitamins

Vitamins are essential micronutrients known for their diverse physiological functions, including antioxidant defense and immune regulation. Vitamin C is a potent antioxidant that scavenges free radicals and regenerates other antioxidants, such as vitamin E. It plays a crucial role in protecting cells from oxidative damage induced by pesticides. Elzoghby et al. (139) evaluated markers of liver function, such as serum levels of ALT and AST, as well as histopathological changes in liver tissues. Rats treated with malathion alone exhibited elevated levels of ALT and AST, indicative of liver damage. However, rats co-administered with vitamin showed reduced levels of ALT and AST, suggesting the protective effects of vitamin C against pesticide-induced hepatotoxicity. Histopathological examination also revealed less severe liver damage (142). Vitamin E comprises a group of fat-soluble antioxidants, including alpha-tocopherol and gamma-tocotrienol. It acts as a lipid-soluble antioxidant, protecting cell membranes from oxidative stress caused by pesticides (143). The experimental study provides evidence supporting the protective effects of vitamin E against organophosphate pesticide-induced oxidative stress in rats. The findings suggest that supplementation with vitamin E enhances the activity of key antioxidant enzymes, such as SOD, CAT, and GPx, thereby mitigating the adverse effects associated with pesticide exposure (144).

Vitamin A and its precursors, such as beta-carotene, possess antioxidant properties that contribute to cellular defense against pesticide-induced oxidative damage (145). Rats treated with atrazine alone showed decreased activities of antioxidant enzymes, such as SOD and catalase, and increased levels of lipid peroxidation, indicative of oxidative stress. In contrast, rats co-administered with vitamin E and atrazine exhibited restored antioxidant enzyme activities and reduced lipid peroxidation levels, suggesting the antioxidant properties of vitamin E (146). Additionally, vitamin A is involved in maintaining epithelial integrity and immune function. Vitamin D regulates calcium and phosphorus metabolism, thereby promoting bone health. Moreover, emerging research suggests its potential role in modulating immune responses and reducing inflammation, which may aid in mitigating pesticide-induced toxicity (147).

### 7.7 Coenzyme Q10

Coenzyme Q10 (CoQ10), also known as ubiquinone, is a naturally occurring molecule present in the body that plays an important role in the creation of cellular energy. CoQ10 is a powerful antioxidant that aids in the neutralization of damaging free radicals and the reduction of oxidative stress. Pesticides can cause free radicals to form in the body, causing cellular damage and inflammation (147). The antioxidant capabilities of CoQ10 may assist to offset these effects and reduce pesticide-induced oxidative stress. CoQ10 is essential in mitochondria, the energy-producing organelles found within cells. Pesticides can interfere with cellular energy generation, causing weariness and tissue damage. CoQ10 supplementation may maintain mitochondrial activity, guaranteeing an appropriate energy supply to cells and maybe assisting cells in coping with pesticide-induced stress. Some insecticides, especially those with neurotoxic characteristics, can cause mitochondrial damage. Because CoQ10 is an essential component of the mitochondrial electron transport chain, it may help shield mitochondria from such damage, sustaining cellular function (148). CoQ10 has been linked to anti-inflammatory properties. Pesticides can cause inflammation in the body, which can lead to tissue damage. CoQ10 has been shown to improve the body’s natural defense systems against pollutants such as pesticides. It may boost the activity of detoxification enzymes in the liver and aid in the elimination of pesticides from the body. CoQ10 has being investigated for its neuroprotective effects. Some pesticides can injure the neurological system, and CoQ10 may provide protection. Some insecticides have been linked to cardiovascular damage. CoQ10 is proven to benefit heart health and may help guard against pesticide-related cardiovascular problems (129). A report provides evidence supporting the potential cardioprotective, antioxidant, and anti-inflammatory effects of coenzyme Q10 against pesticide-induced cardiac toxicity in rats. The findings suggest that coenzyme Q10 supplementation may offer therapeutic benefits in mitigating the adverse effects of pesticide exposure on cardiac health (149).

A report by Mohamed et al. (148) measured SOD activity in rat tissues as an indicator of antioxidant defense. Rats treated with acetamiprid alone showed decreased SOD activity compared to control rats, indicative of reduced antioxidant capacity. However, rats co-administered with curcumin and pesticide exhibited restored SOD activity, suggesting the ability of these phyto-protective nutraceuticals to enhance antioxidant defense against pesticide-induced oxidative stress (150). As an additional indicator of antioxidant defense, CAT activity in rat tissues was evaluated in an earlier report. When compared to control rats, animals treated with deltamethrin alone had lower CAT activity, a sign of compromised antioxidant defense systems. On the other hand, rats who received both the pesticide and rutin concurrently showed increased CAT activity, indicating that these phyto-protective nutraceuticals can improve CAT-mediated antioxidant defense against oxidative stress caused by pesticides (151).

As another indicator of antioxidant defense, GPx activity was also evaluated in rat tissues in previous report. When compared to control rats, animals treated with metribuzin alone had lower GPx activity, a sign of impaired antioxidant defense systems. Rats co-administered with quercetin plus metribuzin, however, showed restored GPx activity, indicating that these phyto-protective nutraceuticals can improve GPx-mediated antioxidant protection against oxidative stress caused by pesticides (152). The findings suggest that supplementation with these phyto-protective nutraceuticals can enhance the activity of key antioxidant enzymes, such as SOD, CAT, and GPx, thereby mitigating the adverse effects associated with pesticide exposure.

The substances curcumin, resveratrol, flavonoids, n-acetylcysteine, omega-3 fatty acids, vitamins, and coenzyme Q10 are highlighted as representative examples due to their relevance in providing protective effects against certain injuries induced by pesticide exposure. These substances have been studied for their potential to counteract the harmful effects of pesticides on the body, such as oxidative stress, inflammation, and cellular damage. By presenting these examples, it is suggested that they may offer promising avenues for mitigating the negative impacts of pesticide exposure on human health (153).

These compounds also possess anti-inflammatory properties, further protecting against pesticide-induced injuries (154). Furthermore, certain nutraceuticals have been found to enhance detoxification mechanisms in the body. Similarly, certain herbs and spices, such as ginger and turmeric, have been found to alleviate pesticide-induced liver and kidney injuries through their antioxidant and anti-inflammatory properties (Table 2).

**TABLE 2:** List of commonly used plants and their medicinal properties against different toxicities.

S No.	Plant	Scientific name	Component(s)	Medicinal properties of nutraceuticals	References
1	Spinach	*Spinacia oleracea*	Leaves	Anticarcinogenic and anti-mutagenic activity	(155)
2	Nettle	*Urtica dioica*	Leaves	Anti-inflammatory, anticancer, and antioxidant activity	(156)
3	Okra	*Abelmoschus esculentus*	Leaves and fruits	Anti-inflammatory and antioxidant property	(157)
4	Drum stick	*Moringa oleifera*	Leaves, seeds, bark, and roots	Antidiabetic, anti-hypertensive, fertility enhancer, antioxidant, and anticancer property	(158)
5	Quinoa	*Chenopodium quinoa*	Seeds	Phenolics and antioxidant property	(159)
6	Inula	*Inula racemosa*	Roots	Antioxidant and anti-bacterial property	(160)
7	Shepherd’s purse	*Capsella bursa-pastoris*	Stem, leaves, and flowers	Antioxidant and anti-inflammatory property	(161)
8	Curry leaves	*Murraya koenigii*	Leaves	Anti-inflammatory, anti-aging, and antioxidant property	(162)
9	Turmeric	*Curcuma longa*	Rhizome	Anti-inflammatory and anticancer property	(163)
10	Black pepper	*Piper nigrum*	Peppercorn	Antioxidant, antimicrobial, anti-inflammatory, antidiabetic, and anticancer property	(164)
11	Sweet pepper	*Capsicum annuum*	Peppercorn	Antioxidant and anti-inflammatory property	(165)
12	Ginger	*Zingiber officinale*	Rhizome	Antioxidant and anti-inflammatory property	(166)
13	Garlic	*Allium sativum*	Bulb, leaves, and flowers	Antioxidant, antidiabetic, anticancer, and anti-inflammatory property	(167)
14	Green tea	*Camellia sinensis*	Leaves	Antioxidant and anticancer property	(168)
15	Red ginseng	*Panax ginseng*	Leaves and roots	Antioxidant property	(169)
16	Purple coneflower	*Echinacea purpurea*	Leaves and flowers	Anti-inflammatory, antioxidant, and antiproliferative property	(170)
17	Fenugreek	*Trigonella foenum-graecum*	Leaves and seeds	Pro-apoptotic and anticancer property	(171)
18	Grapes	*Vitis vinifera*	Fruit	Anti-oxidative, anti-inflammatory, anticancer and anti-aging property	(172)
19	Raspberries	*Rubus idaeus*	Fruit	Antioxidant and anticancer property	(173)
20	Blueberries	*Vaccinium cyanococcus*	Fruit	Antioxidant and anti-proliferative property	(174)
21	Black cumin	*Nigella sativa*	Seeds	Antioxidant property	(175)
22	Fennel seed	*Foeniculum vulgare*	Seeds	Antioxidant property	(176)
23	Kale	*Brassica sabellica*	Leaves	Anticancer, antioxidant, and anti-inflammatory property	(177)
24	Broccoli	*Brassica oleracea*	Stem, leaves, and florets	Anti-proliferative and antioxidant property	(178)
25	Pomegranate	*Punica granatum*	Endocarp	Anticancer, anti-fungal, and anti-inflammatory property	(179)
26	Papaya	*Carica papaya*	Fruit	Antioxidative and anticancer property	(180)
27	Orange	*Citrus sinensis*	Fruit	Antioxidant property	(181)
28	Pineapple	*Ananas comosus*	Fruit	Antioxidant and anticancer property	(182)
29	Apple	*Malus domestica*	Fruit	Antioxidative and anticancer property	(183)
30	Mango	*Mangifera indica*	Fruit	Antioxidant, anti-inflammatory, and anticancer property	(184)
31	Jalapeno	*Capsicum annuum*	Seeds	Antioxidant, anti-obesity, antibiotic, and anticancer property	(185)
32	Amla	*Phyllanthus emblica*	Fruit	Anti-diabetes, anticancer, and antioxidant	(186)
33	Chamomile	*Matricaria chamomilla*	Flowers	Antioxidant and anti-aging properties	(187–189)

## 8 Conclusion

Specific pesticide-mediated toxicities pose significant risks to human health and the environment. Pesticides are widely used in agricultural practices to control pests and increase crop yield, but they can have detrimental effects on nontarget organisms, including humans. However, there is growing evidence that certain food-based nutraceuticals can help mitigate the toxic effects of pesticides. Natural compounds found in various foods, such as antioxidants, vitamins, minerals, and phytochemicals, possess protective properties against pesticide toxicity. These nutraceuticals can act as scavengers of free radicals, reduce oxidative stress, enhance detoxification processes, and boost the immune system. Additionally, they may have the ability to repair damaged DNA and protect vital organs such as the liver and kidneys. Furthermore, incorporating a balanced diet rich in fruits, vegetables, whole grains, and legumes can provide essential nutrients that support overall health and resilience against pesticide toxicities. Additionally, further research is needed to better understand the mechanisms by which nutraceuticals protect against pesticide toxicity and to identify optimal dosages and combinations for effective amelioration.

## Author contributions

MS: Formal analysis, Writing – original draft. SS: Investigation, Methodology, Writing – original draft. SKS: Resources, Writing – review & editing. RB: Formal analysis, Writing – original draft. WA: Resources, Writing – original draft. ASA: Software, Writing – original draft. MA: Formal analysis, Writing – original draft. AA: Methodology, Writing – original draft. EV: Resources, Writing – review & editing. MPS: Project administration, Writing – review & editing.
